# Failed Latarjet procedure: a systematic review of surgery revision options

**DOI:** 10.1186/s10195-021-00587-7

**Published:** 2021-06-21

**Authors:** Matteo Buda, Riccardo D’Ambrosi, Enrico Bellato, Davide Blonna, Alessandro Cappellari, Giacomo Delle Rose, Giovanni Merolla, SICSeG (Italian Society of Shoulder, Elbow Surgery) Research Committee

**Affiliations:** 1 Division of Orthopaedics and Trauma, Madre Teresa Di Calcutta Hospital, Monselice, Padova Italy; 2 IRCCS Orthopedic Institute Galeazzi, Milan, Italy; 3 Department of Surgical Sciences, San Luigi Gonzaga Hospital, University of Turin Medical School, Turin, Italy; 4 Orthopaedic and Traumatology Department, University of Turin Medical School, Turin, Italy; 5 Department of Orthopedics and Orthopedic Oncology, University of Padova, Padova, Italy; 6 Shoulder and Elbow Unit, Humanitas Research Hospital, Rozzano, Milan Italy; 7 Shoulder and Elbow Unit, Cervesi Hospital, Cattolica, AUSL Romagna, Cattolica, Italy; 8 Doctorate School in Clinical and Experimental Medicine, UNIMORE, Modena, Italy

**Keywords:** Failed Latarjet, Eden–Hybinette, Complication, Coracoid transfer, Shoulder stabilization, Recurrent shoulder instability

## Abstract

**Background:**

Revision surgery after the Latarjet procedure is a rare and challenging surgical problem, and various bony or capsular procedures have been proposed. This systematic review examines clinical and radiographic outcomes of different procedures for treating persistent pain or recurrent instability after a Latarjet procedure.

**Methods:**

A systematic review of the literature was performed using the Medline, Cochrane, EMBASE, Google Scholar and Ovid databases with the combined keywords “failed”, “failure”, “revision”, “Latarjet”, “shoulder stabilization” and “shoulder instability” to identify articles published in English that deal with failed Latarjet procedures.

**Results:**

A total of 11 studies (five retrospective and six case series investigations), all published between 2008 and 2020, fulfilled our inclusion criteria. For the study, 253 patients (254 shoulders, 79.8% male) with a mean age of 29.6 years (range: 16–54 years) were reviewed at an average follow-up of 51.5 months (range: 24–208 months).

**Conclusions:**

Eden–Hybinette and arthroscopic capsuloplasty are the most popular and safe procedures to treat recurrent instability after a failed Latarjet procedure, and yield reasonable clinical outcomes. A bone graft procedure and capsuloplasty were proposed but there was no clear consensus on their efficacy and indication.

Level of evidence

Level IV

*Trial registration* PROSPERO 2020 CRD42020185090—www.crd.york.ac.uk/prospero/

## Introduction

The Latarjet procedure is usually recommended for patients with both anterior shoulder instability and bony defects [1]. However, in patients with high functional demands due to their participation in contact sports, coracoid transfer can serve as the treatment of choice when there is no bony defect (71–93% of patients return to their sports following coracoid transfer, as compared to 50–56% who return when treated nonoperatively) [2–5].

Although the Latarjet procedure is a safe and effective technique for managing anterior instability, rates of recurrence have been reported to be between 7.5 and 11.6% [6, 7]. Moreover, earlier studies demonstrated considerable reoperation rates of 14%, with a high prevalence in the first postoperative year (73%) [8].

**Fig. 1:**
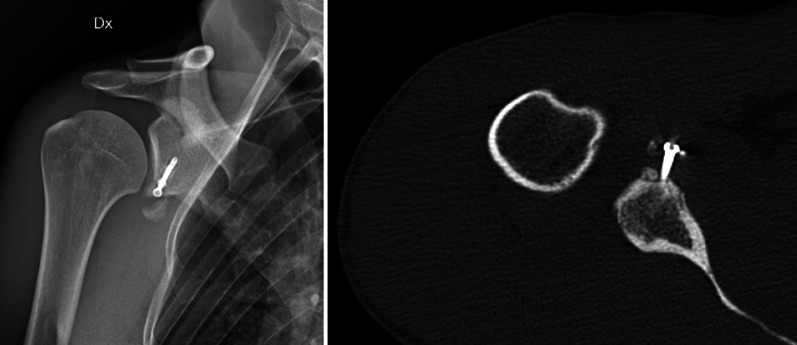
Graft lysis and partial dislocation

Latarjet is a technically demanding procedure, and technical mistakes—whether associated with biological factors or not—can contribute to the risk of failure. An incorrect initial diagnosis, bone block or hardware malpositioning, misdiagnosis of associate lesions (e.g. a Hill–Sachs lesion, posterior capsular labral lesion, SLAP lesion, etc.), bone graft lysis (Fig. 1), nonunion with coracoid migration, an overly accelerated and incorrect rehabilitation regime, subjective laxity and new trauma are reported to be risk factors for instability recurrence or persistent pain [9, 10].

Recurrent anterior shoulder instability or persistent pain after the Latarjet procedure remains a surgical challenge; however, it can be successfully treated with all-arthroscopic or open procedures [11].

The purpose of this systematic review was to evaluate clinical and radiographic outcomes, the rate of failure due to recurrence or loss of stability, complications (including osteoarthritis following screw impingement, loosening or breakage) and the rate of return to sport in patients who undergo revision surgery after a failed Latarjet procedure.

## Methods

### Data search protocol

**Fig. 2:**
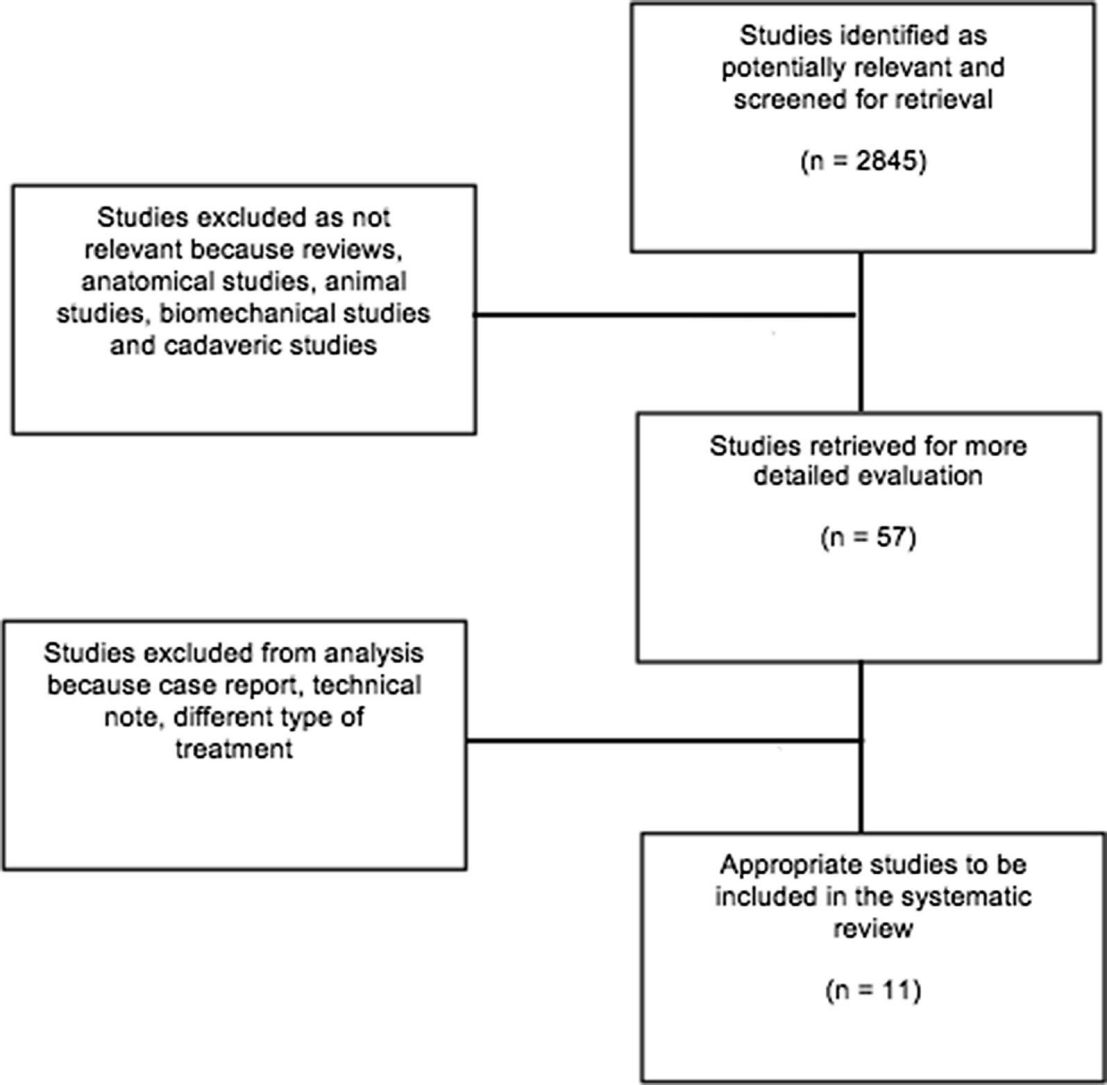
PRISMA flow chart

A systematic review of the existing literature was performed to identify all studies dealing with a failed Latarjet procedure. The Preferred Reporting Items for Systematic Reviews and Meta-Analyses (PRISMA) guidelines were followed for article identification [12]. The search algorithm, derived from the PRISMA guidelines, is shown in Fig. 2. The research was performed using the MEDLINE, Scopus, CINAHL, Embase and Cochrane databases up to December 2020. The review was registered on the PROSPERO database (CRD42020185090). The leading search items were “failed” OR “failure” OR “revision” AND “Latarjet” OR “shoulder stabilization” OR “shoulder instability”. The complete search strategy is shown in Table 1. Additionally, the reference lists of the selected articles were screened for further relevant publications.

**Table 1:** Inclusion and exclusion criteria

Database	Medline, Cochrane, EMBASE, Google Scholar and Ovid
Date	December 2020
Language accepted	English
Keywords matched	“Eden-Hybinette” OR “Complication” OR “Recurrent shoulder instability” AND “Failed Latarjet” OR “Coracoid transfer” OR “Shoulder stabilization”
Type of articles excluded	Reviews, case reports, animal studies, cadaver studies, biomechanical studies, tumoral studies, technical notes
Inclusion criteria	Surgical treatments of a failed Latarjet procedure with preoperative and postoperative outcomes of the patients (using outcome scores, measuring ROM); description of the follow-up period; detailed information on complications and their management
Exclusion criteria	Studies on Latarjet as the primary surgical intervention, follow-up period shorter than 12 months; no information on surgical intervention, complications, clinical outcomes, radiographic outcomes and statistical analysis of the relative results

### Study selection and eligibility criteria

We conducted a systematic review of all Level I–IV studies published in English from January 1990 to December 2020 (according to the 2011 Oxford Levels of Evidence) [13]. The articles were analysed regardless of their title and abstract by two independent investigators (M.B. and R.D.). If a disagreement arose, the two investigators conducted a discussion until they reached a consensus. Articles reporting clinical and/or radiological outcome data in patients surgically treated for failure of the Latarjet procedure were included; case series with less than five cases, case reports, editorials, systematic reviews and meta-analyses were excluded.

### Data extraction, synthesis and analysis

The reviewers analysed all the information available from the articles (data, type of study, level of evidence, demographic data, diagnosis, type of surgical procedure, follow-up duration, outcomes and complications) and entered it into a spreadsheet for analysis.

### Assessment of the quality of the article

Studies were evaluated for methodological research quality using the Modified Coleman Methodology Score (MCMS) criteria [14]. Each study was assessed to give a total score ranging from 0 to 100 points. A score of 100 indicates that the study largely avoids chance, various biases and confounding factors. The final score was defined as excellent if it was between 85 and 100 points, good if it was between 70 and 84 points, fair if it was between 50 to 69 points and poor if it was < 50 points. Results are reported in Table 2.

**Table 2:** Coleman score results

Article	Part A							Part B		
	Study size: number of patients	Mean follow-up	Surgical approach	Type of study	Diagnostic certainty	Description of surgical technique	Rehabilitation protocol	Outcome criteria	Procedure for assessing outcomes	Description of subject selection process
Flurin et al. (2020) [15]	4	7	0	0	5	10	0	7	8	5
Boileau et al. (2019) [20]	0	4	10	10	5	10	5	7	8	5
Khan et al. (2019) [21]	0	7	0	0	5	0	0	0	8	5
Provencer et al. (2019) [22]	4	7	10	10	5	10	0	7	8	5
Lavoue et al. (2019) [16]	4	10	10	0	5	10	5	7	8	10
Willemot et al. (2018) [17]	0	7	0	0	5	0	0	7	8	5
Giannakos et al. (2017) [23]	0	4	10	0	5	10	5	7	3	0
Cuellar et al. (2016) [24]	0	4	10	0	5	10	0	7	8	5
Castagna et al. (2010) [25]	0	7	0	0	5	10	5	7	8	5
Boileau et al. (2009) [18]	0	10	0	10	5	10	5	7	8	5
Lunn et al. (2008) [19]	4	10	10	0	5	10	0	7	3	5

## Results

### Literature review

During the first electronic search, we identified 2845 relevant publications. After the application of the inclusion criteria, 57 studies remained. Of these, 46 studies were excluded because they were case reports, technical notes or did not meet the inclusion criteria. Eleven studies ultimately met the inclusion criteria, five were retrospective [15–19], and six were case series [20–25].

### Demographics

The total number of patients was 253 (254 shoulders, range: 7–46), with 202 males (79.8%, range: 59–100%). The mean age was 29.6 years (range: 16–54), with an average follow-up of 51.5 months (range: 24–208). The mean time from initial surgery to recurrence episode was 32.9 months (range: 1–318 months), while the mean time from initial surgery to revision surgery was 49.2 months (range: 2–336 months). Demographic data are reported in Table 3.

**Table 3:** Demographic data

Author (year)	Study type	Patients	Male, mean (%)	Age, mean (years) ± SD (range)	Follow-up, mean (months) ± SD (range)	Surgical technique *n* (%)	Associated procedure, *n* (%)	Time to recurrence, mean (months) ± SD (range)	Time to revision, mean (months) ± SD (range)
Flurin et al. (2020) [15]	RS (multicentre)	46	38 (83)	28.8 (17–53)	38	E-H procedure	38 (83) capsuloplasties 8 (17) notch filling procedures	46 (1–318)	N/A
Boileau et al. (2019) [20]	CS	7	5 (71.4)	30.7 (17–47)	18 (24–72)	Arthroscopic E-H + capsulorrhaphy	3 (43) Hill–Sachs remplissages	18.7 (3–60)	33 (12–72)
Khan et al. (2019) [21]	CS	16	12 (75)	27.4 ± 6.7 (16–41)	N/A	7 (44) E-H procedures 7 (44) arthroscopic stabilizations 1 (6) E-H + arthroscopic stabilization 1 (6) arthroscopic remplissage	None	N/A	8.7 ± 7.1 (2–26)
Provencer et al. (2019) [22]	CS	31	31 (100)	25.5 (19–38)	47 (36–60)	Open fresh DTA fixation	DTA soaked in 10 min PRP	N/A	N/A
Lavoue et al. (2019) [16]	RS	41	33 (80)	29 (16–46)	72 (24–208)	22 (54) arthroscopic unipolar stabilizations 19 (46) arthroscopic bipolar stabilizations	28 (68) hardware removal procedures 5 (12) biceps tenodeses 2 (5) glenoidoplasties	28 (1–240)	
Willemot et al. (2018) [17]	RS	26	20 (77)	29.4 ± 6.6	43.7 ± 27.7	20 (76.9) E-H procedures 3 (11.5) reimplantations of coracoid graft with iliac crest bone or autologous cancellous bone grafts 3 (11.5) graft repositioning procedures	None	N/A	37.2
Giannakos et al. (2017) [23]	CS	12	9 (75)	37.5 (26–54)	28.8 (15–60)	Arthroscopic E-H procedure	4 (30) brachial plexus releases	N/A	N/A
Cuellar et al. (2016) [24]	CS	12	10 (83)	32.2 (27–36)	36 (24–96)	Arthroscopic capsule plication	3 (25) posterior labrum/capsular repairs 5 (42) screw removals	N/A	N/A
Castagna et al. (2010) [25]	CS	17 (18 shoulders)	N/A	33.5 ± 10.6 (20–53)	69 (24–108)	Arthroscopic capsulorrhaphy	2 (11) SLAP repairs 9 (50) multiple capsular plications 1 (6) screw removal	N/A	81 (12–336)
Boileau et al. (2009) [18]	RS	19/22 (11 Latarjet, 5 E-H , 3 open Bankart, 3 capsular shifts)	17 (77)	31 ± 6.1	43 ± 17	Arthroscopic Bankart repair	12 (54) inferior capsular plications 4 (18) rotator interval closures 8 (50) screw removals 1 (4.5) rotator cuff repair 1 (4.5) biceps tenotomy 2 (9) type IV SLAP lesion resections	25 ± 15.2	N/A
Lunn et al. (2008) [19]	RS	34	27 (59)	30 (18.4–51.8)	69.6 (24–206.4)	E-H procedure	16 (47) capsule sutures 4 (12) graft placements above the intact coracoid 5 (15) conjoint tendon sutures 5 (15) subscapularis repairs	26.5 (1–204)	64.8

*RS* retrospective study, *CS* case series,* E-H* Eden–Hybinette, *DTA* soaked in 10 min PRP, *PRP* platelet-rich plasma

### Indications

Indications for the revision of a Latarjet procedure were persistent pain or recurrent anterior instability, defined as at least one episode of dislocation or subluxation and a minimum follow-up of 24 months.

Humeral or glenoid bone defects were not considered a cut-off criterion to exclude patients, except in the study reported by Cuellar et al., who excluded patients with glenoid bone defects > 25% [24].

### Surgical technique

Different surgical techniques were reported in the articles selected for this review and were performed according to the surgeon’s preferences and experience. Arthroscopic Eden–Hybinette was performed in two studies (19 patients, 7.4%) [20, 23] and open Eden-Hybinette in four studies (108 patients, 42%) [15, 17, 19, 21]. A tricortical graft was harvested from the ipsilateral side and was fixed with titanium screws. A one or two suture-button device was used to fix the graft in only one study [20].

Open stabilization with fresh distal tibial allograft (fixed in place with two 4.0-mm, fully threaded, noncannulated bicortical interference screws) was performed in one study (31 patients, 12.1%) [22]. Arthroscopic capsuloplasty was performed in five studies (97 patients, 38.1%) [16, 18, 21, 24, 25].

Different associated procedures were performed during the revision surgery; they are summarized in Table 3.

### Surgical treatments associated with a primary bone block procedure

A total of 139 associated procedures were performed during the index revision surgery. Capsuloplasties associated with bone block fixation were performed in 75 patients [15, 18, 19, 25], a biceps tenodesis was performed in five patients [16], a biceps tenotomy in one patient [18], a glenoidoplasty in 10 patients [15, 16], Hill–Sachs remplissage with bone block fixation in three patients [20], Hill–Sachs remplissage during a capsuloplasty in 19 patients [16], brachial plexus release in four patients [23], posterior labrum and/or capsular repair in three patients [24], SLAP lesion repair in four patients [18, 25], rotator cuff repair in six patients [18, 19], conjoint tendon suture in five patients [19] and rotator interval closure in four patients [18]. Surgical techniques associated with bone block fixation are described in Table 3.

### Clinical and functional outcomes

All clinical scores improved after surgery. Clinical outcomes were assessed using the Constant and Murley Score (CS) before and after surgery in three studies [20, 24, 25], the Walch–Duplay score in seven studies [15–20, 23], the Rowe score in eight studies [15, 16, 18–20, 23–25], the American Shoulder and Elbow Surgeons Shoulder Score (ASES) in two studies [22, 25], the Single Assessment Numeric Evaluation (SANE) in one study [22], the University of California, Los Angeles Shoulder Score (UCLA) in two studies [18, 25], Western Ontario Shoulder Instability index (WOSI) in three studies [17, 22, 23], the Subjective Shoulder Value (SSV) in two studies [16, 20], the Visual Analogue Scale (VAS) for pain in four studies [16, 18, 24, 25] and ROM evaluation in six studies [16, 18, 19, 22–24]. Details from the included articles are provided in Table 4.

**Table 4:** Clinical results

Author (year)	Preoperative results	Preoperative imaging results, *n* (%)	Postoperative care	Clinical results	Return to sport	Imaging results, *n* (%)	Complications
Flurin et al. (2020) [15]		9 (20) showed a graft fracture 12 (26) showed complete graft lysis 12 (26) showed partial graft lysis 3 (6.5) showed S–P grade 1–2 osteoarthritis	Immobilization for 6 weeks in 45% of patients, 4 weeks in 21% and 3 weeks in 9%	Stability = 86% Satisfaction = 80% Rowe score = 76 Walch-Duplay score = 68	60% resumed sports; 19.5% to the same level 19.5% to a lower level 21% changed to another sport	40 (86) showed graft union 9 (20) showed partial graft lysis 3 (7) showed complete graft lysis 2 (4) showed S–P grade 1–2 osteoarthritis 2 (4) showed S–P grade 3–4 osteoarthritis	6 recurrences of instability 1 ulnar nerve impingement related to the immobilization 1 infection (*Cutibacterium acnes*) 1 bone block fracture revised by a second Eden–Hybinette procedure
Boileau et al. (2019) [20]	Pain score = 7 CS = 32 SSV = 31% SSV for sport = 10%	5 (71) showed graft malpositioning 5 (71) showed graft nonunion 6 (86) showed partial resorption 3 (43) showed screw mobilization 3 (43) showed a broken screw	Neutral rotation sling for 2 weeks Self-rehabilitation with pendulum exercises at 2 weeks Rehabilitation with physiotherapist at 4 weeks Heavy lifting at 12 weeks Return to sports at 3–6 months	Pain score = 2.4 Constant score = 81.4 SSV = 87% (*P* < .001) SSV for sport activities = 70% (*P* < .001) Walch-Duplay = 85.7 (65–100) Rowe scores = 86.4 (70–100) FF = 176 (150–180) ER = 56 (0–90)		Complete healing at 6 m 2 (29) showed S–P grade 1–2 osteoarthritis	3 showed hypoesthesia of the iliac crest
Khan et al. (2019) [21]			Sling, active-assisted FF and ER for 3 w after active ROM Strengthening exercises at 6 weeks	83.3% success rate after Eden-Hybinette 77.8% success rate after arthroscopic approach 100% success rate after arthroscopic posterior stabilization	7/11 (64%) returned to the same level of sport	N/A	
Provencer et al. (2019) [22]	11 (35.5%) showed recurrent shoulder dislocation 20 (64.5%) showed recurrent subluxation FF = 152 (125 -170) ABD = 110 (70 -160) ER = 22.5 (10 -50) ASES 40 ± 6.8 (10–70) SANE 44 ± 7.2 (20–55) WOSI 1300 ± 237 (1050–1995) WOSI % normal 38.1 ± 11.3 (5–50)	24 (78) showed complete graft lysis S–P = mean 0.5 (0–3) Mean glenoid bone loss of 30.3% (25–49%)		FF = 161° (140–175°, *P* = .001) ABD = 138° (110–160°, *P* = .001) ER = 37.6° (25–55°, *P* = .001) ASES = 92 ± 2.2 (85–97, *P* = .001) SANE = 91 ± 5.0 (80–100, *P* = .001) WOSI = 310 ± 111 (42–630, *P* = .001) WOSI % normal = 85.3 ± 5.3 (70–98, *P* = .001)		28 (90) showed graft union 3 (10) showed partial graft healing 24 (77) showed partial graft lysis	None
Lavoue et al. (2019) [16]	VAS = 5.8 ± 2 (0–9) SSV = 51% ± 19 (5–90) SSV sport = 44% ± 27 (0–80) Rowe = 54 ± 28 (5–60) Walch-Duplay = 17 ± 19 (−10–55) FF = 168° ± 18° (90–180°) ER = 53° ± 21° (20–100°)	11 (27) showed S–P grade 1–3 osteoarthritis 20 (49) graft nonunions 1 (2) showed complete graft lysis 11 (27) showed graft malpositioning	Neutral rotation sling, passive mobilization and pendulum exercise for 4 weeks Active physiotherapy at 4 weeks Return to sports at 3–6 m	VAS = 1.3 ± 2 (0–7) (*P* = .0001) SSV = 83% ± 18 (20–100) (*P* = .0001) SSV sport = 69% ± 24% (5–100%) (*P* < .05) Rowe = 78 ± 24 (10–100) (*P* < .05) Walch–Duplay = 76 ± 28 (−5 to 100) (*P* < .05) FF = 172° ± 15° (110–200°) (*P* > .05) ER = 58° ± 21° (10–90°) (*P* = .95) 34 (83%) were satisfied or very satisfied	29/36 (81%) patients returned to sport 16 (55%) returned to traumatic/overhead sport	23 (57) showed S–P grade 1–3 osteoarthritis (*P* = .02)	5 recurrences of instability
Willemot et al. (2018) [17]		11 (42) showed graft nonunion 6 (23) showed graft lysis 4 (15) showed graft/hardware malpositioning 5 (19) showed fracture and graft migration 7 (27) showed S–P grade 1 osteoarthritis 4 (15) showed S–P showed grade 2 osteoarthritis 3 (12) showed S–P grade 3 osteoarthritis		SSS = 60.2% ± 19.6% WOSI scores = 709.3 ± 412.5 points	9 (46.1%) returned to prerevision level of sport	12 (46) showed S–P grade 1 osteoarthritis 3 (12) showed S–P grade 2 osteoarthritis 5 (19) showed S–P grade 3 osteoarthritis	3 recurrences of instability/subluxations
Giannakos et al. (2017) [23]	Rowe score = 16.25 ± 11.10 Walch–Duplay = 11.76 ± 17.10	4 (34) showed graft malpositioning 3 (25) showed graft nonunion 9 (75) showed S–P grade 1–2 osteoarthritis 3 (25) showed S–P grade 3–4 osteoarthritis	ABD pillow with pROM for 3 weeks Active-assisted exercises at 3 weeks Strengthening exercises at 6 weeks Return to sports at 3 months	Walch–Duplay = 77 ± 22.7 (*P* < .0001) Rowe = 78 ± 23.5 (*P* < .0001) WOSI = 603 ± 399 Satisfaction = 67% Stability = 83%	58% resumed sports; 33% at a lower level	7 (58) showed graft union 4 (33) showed graft nonunion 1 refused postoperative CT scan 8 (67) showed S–P grade 1–2 osteoarthritis 4 (33) showed S–P grade 3–4 osteoarthritis	4 arthroscopic hardware removals due to possible impingement with the humeral head 1 persistent brachial plexus neuropathy 1 screw breakage
Cuellar et al. (2016) [24]	12 (100%) showed inferior and/or anteroinferior apprehension, Gagey and jerk tests + contralateral hypermobility signs 2 showed polyarticular laxity signs 3 showed hypermobility in the posterior direction 7 showed drive-through sign (grade 2–3) ER = 51.3° (45–55°) CS = 44.9 ± 7.10 Pain score 2.38 ± 1.06 ADL = 8.9 ± 4.58 ROM = 16.8 ± 5.23 Strength during weight lifting = 16.9 ± 2.59 Rowe = 49.5 ± 10.1 VAS = 6.75 ± 1.17	5 (42) showed a loose or broken screw 2 (17) showed graft nonunion 2 (17) showed graft lysis 1 (8) showed S–P grade 1–2 osteoarthritis 2 (17) showed S–P grade 3–4 osteoarthritis		CS = 89.3 ± 12.6 (*P* < .0001) Pain score = 14.1 ± 2.48 (*P* < .0001) ADL = 18.5 ± 3.86 (*P* < .001) ROM = 33.5 ± 9.38 (*P* < .0001) Strength during weight lifting = 23.1 ± 3.72 (*P* < .01) Rowe score = 80.9 ± 10.9 (*P* < .0001) VAS score = 1.38 ± 1.06 (*P* < .0001)		N/A	None
Castagna et al. (2010) [25]		1 (6) showed degenerative bipolar arthritis	Sling and pendulum exercises for 1 month Passive ROM at 1 month, avoiding forced ABD and ER Active mobilization at 2 months Return to contact sports at 6 months	CS = 78.4 ± 16.2 (40–100) UCLA = 27.2 ± 6.9 (10–35) ASES = 99.6 ± 14.7 (73–120) Rowe score = 75.2 ± 25.3 (0–100) VAS score = 2.9 ± 3.7 (0–9)	11 (61%) returned to previous sporting/working activities	N/A	3 recurrences (1 dislocation and 2 with subluxation/spraining)
Boileau et al. (2009) [18]	3 (14%) dislocation 12 (55%) subluxation 7 (32%) both Walch–Duplay = 13.8 ± 17 Rowe = 15 ± 19 UCLA = 20.9 ± 6 Pain = 2.8	2 (9) showed a malunited glenoid fracture 6 (27) showed anteroinferior mild glenoid erosion 17 (77) showed a Hill–Sachs lesion 9 (41) showed graft malpositioning 3 (14) showed graft lysis 3 (14) showed graft fracture 1 (5) showed graft nonunion 3 (14) showed S–P grade 1–2 osteoarthritis	IR immobilization and pendulum exercises for 4 weeks. =Rehab with physiotherapist at 1 month (FF and ER limited to 45° until the 45th day) Return to sports at 6 months	Walch–Duplay = 85 ± 21 (*P* < .0001) Rowe = 81 ± 23 (*P* < .0001) UCLA = 29.5 ± 7 (*P* < .0001) Pain = 1.1 (*P* < .039) Subjective shoulder value score = 83 ± 23% (50–100%) 17 (89%) very satisfied or satisfied, 1 unhappy	9 (47%) returned to the same level of sport All returned to previous occupation	5 (26) showed S–P grade 1–2 osteoarthritis	1 showed sympathetic dystrophy
Lunn et al. (2008) [19]	FF = 170 (165–180) ER = 70 (30–100) Walch–Duplay score = 3 type 1, 14 type 2, 6 type 3, and 6 type 4	12 (35) showed graft malpositioning 13 (38) showed graft lysis 4 (12) showed S–P grade 1–2 osteoarthritis 2 (6) showed S–P grade 3–4 osteoarthritis		13 (38%) showed apprehension sign + subjective scoring = 20 (59%) excellent, 10 (29%) good, 3 (9%) fair, 1 (3%) poor Walch–Duplay = 78 ± 21.3 Rowe = 82 ± 17.5 Satisfaction = 88% Stability = 88%	94% resumed sports; 62% to the same level 32% to a lower level	6 (17) showed graft lysis 4 (12) showed S–P grade 1–2 osteoarthritis 6 (18) showed S–P grade 3–4 osteoarthritis	4 recurrences of instability 5 showed discomfort or hypoesthesia at the harvest site 1 superficial wound infection

*E-H* Eden–Hybinette, *WOSI* Western Ontario Shoulder Instability index, *SSS* Subjective Shoulder Score, *CS* Constant score, *S–P* Samilson and Prieto, *ROM* range of motion, *FF* forward flexion, *ER* external rotation, *IR* internal rotation, *VAS* visual analogue scale, *SSV* Subjective Shoulder Value, *UCLA* University of California, Los Angeles Shoulder Score, *ASES* American Shoulder and Elbow Surgeons Shoulder Score, *SANE* Single Assessment Numerical Evaluation, *DTA* distal tibial allograft, *SLAP* superior labral tear from anterior to posterior

### Cause of failure and preoperative imaging evaluation

A trauma after the index procedure was the trigger for instability in 60 patients [15, 16, 18, 19, 23, 25]. Minor or moderate trauma was the cause of recurrent instability in 15 patients [15, 25]. An epileptic seizure relapse was reported in only one case [19].

Preoperative imaging evaluation showed 42 cases of graft nonunion [16–18, 20, 23, 24], 45 cases of graft/hardware malpositioning [16–20, 23], 17 cases of graft fracture [15, 17, 18], 61 cases of complete graft lysis [15–19, 22, 24], 18 cases of partial graft lysis [15, 20], three cases of screw loosening [20], eight cases with a broken screw [20, 24], 17 cases with a Hill-Sachs lesion [18] and two cases of malunited glenoid fracture [18].

Samilson and Prieto grade 1–2 osteoarthritis was diagnosed in 39 patients [15–19, 23–25], while Samilson and Prieto grade 3–4 osteoarthritis was diagnosed in 14 patients [17, 19, 23, 24]. Only one study did not report preoperative imaging [21].

### Immobilization and rehabilitation

The authors suggested the use of a neutral shoulder sling for 2 weeks in one study [20], for 3 weeks in three studies [15, 21, 23], for 4 weeks in three studies [15, 16, 25] and for 6 weeks in one study, and there were no substantial differences in outcome between capsuloplasty and the Eden–Hybinette procedure [15]. Boileau et al. [18] suggested that internal rotation immobilization should be implemented for 4 weeks and pendulum exercises from the first postoperative day. Most authors encouraged immediate passive ROM exercises of the elbow and wrist and early passive pendular exercises to reduce inflammation and prevent shoulder stiffness. In most cases, active-assisted exercises were started after 3 weeks and strengthening exercises were introduced at 6 weeks. The patient was allowed to return to sport after 3–6 months. Four studies did not report a postoperative protocol [17, 19, 22, 24]. The rehabilitation protocols are summarized in Table 4.

### Postoperative imaging evaluation

Postoperative imaging was evaluated in eight studies (208 patients) [15–20, 22, 23]. Of the 156 patients treated with bone grafting, nonunion was observed in 10 patients [15, 23], graft lysis in 21 patients (complete lysis in three and partial lysis in 18 shoulders) [15, 19, 22], and partial graft healing was seen in three patients [22].

Glenohumeral osteoarthritis progression was observed in 76 (36.5%) patients an average of 51.7 months after the revision procedure (Samilson–Prieto stage 1–2 in 33 (15.9%) patients and stage 3–4 in 43 (20.7%) patients) [15–20, 23].

### Return to sports

One hundred and ninety-four patients practised sporting activities before their injuries. After revision surgery, 99 (51%) returned to their preinjury level [15–19, 21, 23, 25] while 25 (6.5%) patients returned to a lower level [15, 19, 21, 23]. Seven patients in one study had to change their sporting activities [15].

### Recurrence, complications and reoperations

The overall rate of recurrence and complication was 17.3% (44/254 patients), and included 22 cases of recurrence (8.6%) [15–17, 19, 21, 25], one ulnar nerve impingement related to the immobilization (0.3%) [15], three *Cutibacterium acnes* infections (1.2%) [15, 19, 21], one bone block fracture revised by a second Eden–Hybinette procedure (0.3%) [15], eight cases of hypoesthesia in the skin area of the iliac crest where harvesting was performed (3.1%) [19, 20], one transient ilioinguinal nerve injury (0.3%) [21], one case of infected graft fracture and screw loosening (0.3%) [21], four arthroscopic hardware removals due to possible impingement with the humeral head (1.5%) [23], one persistent brachial plexus neuropathy (0.3%) [23], one screw breakage (0.3%) [23] and one case of sympathetic dystrophy (0.3%) [18].

### Quality assessment

The mean value of the Coleman score was 51 points (range: 32–69), showing that the mean quality of the included studies was fair (Table 2). Inter-rater agreement failed to show a significant difference in Coleman score mean values.

## Discussion

The most important finding from our study was that Eden–Hybinette (with an iliac bone graft or a distal fresh allograft) and capsuloplasty are the most popular and safe procedures to treat recurrent instability after a failed Latarjet procedure, with good to excellent clinical outcomes and satisfaction rates reported (ranging between 67 and 89%). However, we found that the studies we analysed lacked a common consensus regarding when to choose a bone block procedure or capsuloplasty, which may be due to differences in the skills and experience levels of the surgeons in the various studies. Nevertheless, the Eden–Hybinette procedure is preferred by most authors when the failure is due to complications of the coracoid graft.

Although the Latarjet procedure is an effective surgical technique to treat recurrent anterior shoulder instability whether or not glenoid bone deficiency is present, substantial complications are reported to occur in 30% of cases [8].

We found that revision procedures had similar (relatively low) rates of major postoperative complications to index Latarjet procedures. Overall, in our analysis, the recurrence rate of instability was 8.6%. Hurley et al. reported similar results with a recurrent instability rate of 8.5% in 822 patients treated with Latarjet procedure (3.2% of patients with recurrent dislocations) [26].

Hurley et al. reported a high overall rate of return to play after the Latarjet procedure (88.8% of patients returned to play, with 72.6% returning to the same level of play) [27]. However, almost one-fifth of athletes were not able to return to the same level. Our rates were lower: 99 (51%) out of 194 patients who performed a sporting activity returned to the same level, while 25 (6.5%) patients returned to a lower level.

In the literature, the rate of new signs or progression of radiographic arthritis was described as being between 28 and 38.2% of patients treated with primary open Latarjet [26, 28]. These data are in line with those for the patients analysed in our study. At a mean follow-up of 51.7 months following the revision surgery, we found a relatively similar incidence of glenohumeral osteoarthritis progression (76 patients among the 208 analyzed; 20.7% with Samilson–Prieto stage 3–4 osteoarthritis).

Keeping in mind that recurrent instability after the Latarjet procedure usually occurs within the first few postoperative years (73%), the most frequent causes are technical mistakes or biological factors [8].

In our analysis, 10 studies reported preoperative imaging. Latarjet revision was due to nonunion or complete graft lysis in 42 (17.6%) and 61 (25.6%) patients, respectively, while graft malpositioning was found in 45 (18.9%) patients.

Although the ipsilateral iliac crest remains the leading autograft donor site, different allograft donor sites have been proposed, such as the femoral head, the humeral head, the glenoid and the distal tibia [29]. These techniques reduce the risk of discomfort or hypoesthesia at the harvest site on the iliac crest and reduce surgical time, albeit at the expense of higher costs and a lack of availability [30].

Provencer et al. suggest using fresh distal tibia augmentation as a viable and highly effective bone graft to restore the glenoid area [22]. The distal tibia has a similar radius of curvature and similar articular cartilage to the glenoid, so it is used as an allograft in settings where the Latarjet procedure is not optimal, including cases in which more than 30% of the glenoid width has been lost, and in cases where the coracoid is absent owing to prior surgery or trauma [31].

Most of the grafts were open procedures. The arthroscopic technique could offer the opportunity to be more precise and increase the accuracy of graft positioning, even though its superiority has not yet been demonstrated [32, 33]. In addition, the arthroscopic approach provides the ability to explore the brachial plexus in the setting of a revision procedure, thus reducing the risk of neurological damage through improved visualization during the bone grafting procedure [16, 23].

Associated lesions such as a posterior or anterior capsule-labral lesion, a SLAP lesion, long head of biceps pathologies, an engaging Hill–Sachs lesion or a rotator cuff tear can provoke recurrent instability or persistent pain if they are not correctly repaired [34].

On the other hand, disadvantages of the arthroscopic bone grafting procedure are a considerable learning curve, higher costs and a longer average surgical time [35].

Cadaveric studies have reported contrasting results regarding whether capsular repair significantly increases the stabilizing effect of the Latarjet procedure [36–38]. Suturing the capsule to the coracoacromial ligament seems to have a protective effect (23% of the resistance) on the translational forces in the end-range arm position against anterior subluxation or dislocation of the humeral head [36]. For this reason, beyond its capacity to repair a misdiagnosed or untreated associated lesion and address the graft or hardware positioning of the index procedure, the application of arthroscopic capsuloplasty to treat a failed Latarjet procedure can be beneficial for subjects who complain of instability at the end-range arm position [34]. On the other hand, arthroscopic capsular repair is not recommended in patients with severe glenoid bone loss [16].

Finally, factors such as the age of the athlete, their participation in a contact sport, the presence of a concomitant Hill–Sachs lesion, the number of dislocations and the number of operations that the patient has undergone must be taken into consideration before performing revision with capsular stabilization [39, 40].

## Limitations

Limitations of the present systematic review are mainly related to the low quality and quantity of the studies available in the literature; all the studies included in this work were Level IV case series. Moreover, most of the studies did not specify their inclusion criteria regarding bone loss on the glenoid and humeral sides. In our opinion, such criteria are important for setting the correct indication for a bone block stabilization or capsuloplasty. In addition, the recurrence and complication rates may depend on the type of technique used and the skills and experience of the surgeon performing the surgery.

## Conclusions

Eden–Hybinette with an iliac bone graft and capsuloplasty are the most popular and safe procedures to treat recurrent instability after a failed Latarjet procedure; they are reported to produce reasonable clinical outcomes and satisfaction. However, the proportion of patients who return to sporting activity is lower when compared to the index procedure.

There is no clear consensus among surgeons regarding when a bone graft or capsuloplasty should be performed. When conservative treatment fails, it is crucial to identify all possible causes of failure before deciding upon the correct surgical revision.

Generally, graft failure was treated with graft substitution through either an open or arthroscopic Eden–Hybinette procedure; on the other hand, when the graft was well positioned, arthroscopic capsuloplasty was preferred. Further comparative studies are needed to clarify the potentially promising superiority of and the correct indication for one technique compared to the others, especially when recurrent anterior instability persists in patients who do not suffer complications from a well-positioned graft.

## Data Availability

All data and materials can be retrieved from the references and articles included in the systematic review.
